# Social determinants of breast cancer in the Caribbean: a systematic review

**DOI:** 10.1186/s12939-017-0540-z

**Published:** 2017-04-05

**Authors:** Catherine R. Brown, Ian R. Hambleton, Shawn M. Hercules, Miriam Alvarado, Nigel Unwin, Madhuvanti M. Murphy, E. Nigel Harris, Rainford Wilks, Marlene MacLeish, Louis Sullivan, Natasha Sobers-Grannum, The U.S. Caribbean Alliance for Health Disparities Research Group (USCAHDR), M. Alvarado, N. Bennet, A. Bidulescu, CR. Brown, T. Ferguson, D. Francis, I. R. Hambleton, E. N. Harris, C. Hassell, A. J. M. Hennis, SM. Hercules, C. Howitt, M. MacLeish, MM. Murphy, T. A. Samuels, N. Sobers-Grannum, L. Sullivan, N. Unwin, R. Wilks, L. Williams, N. Younger-Coleman

**Affiliations:** 1 Chronic Disease Research Centre, Bridgetown, Barbados; 2 The University of the West Indies, Cave Hill, Barbados; 3 The University of the West Indies, Kingston, Jamaica; 4 Sullivan Alliance, Alexandria, VA USA

## Abstract

**Background:**

Breast cancer is the leading cause of cancer deaths among women in the Caribbean and accounts for >1 million disability adjusted life years. Little is known about the social inequalities of this disease in the Caribbean. In support of the Rio Political Declaration on addressing health inequities, this article presents a systematic review of evidence on the distribution, by social determinants, of breast cancer risk factors, frequency, and adverse outcomes in Caribbean women.

**Methods:**

MEDLINE, EMBASE, SciELO, CINAHL, CUMED, LILACS, and IBECS were searched for observational studies reporting associations between social determinants and breast cancer risk factors, frequency, or outcomes. Based on the PROGRESS-plus checklist, we considered 8 social determinant groups for 14 breast cancer endpoints, which totalled to 189 possible ways (‘relationship groups’) to explore the role of social determinants on breast cancer. Studies with >50 participants conducted in Caribbean territories between 2004 and 2014 were eligible for inclusion. The review was conducted according to STROBE and PRISMA guidelines and results were planned as a narrative synthesis, with meta-analysis if possible.

**Results:**

Thirty-four articles were included from 5,190 screened citations. From these included studies, 75 inequality relationships were reported examining 30 distinct relationship groups, leaving 84% of relationship groups unexplored. Most inequality relationships were reported for risk factors, particularly alcohol and overweight/obesity which generally showed a positive relationship with indicators of lower socioeconomic position. Evidence for breast cancer frequency and outcomes was scarce. Unmarried women tended to have a higher likelihood of being diagnosed with breast cancer when compared to married women. While no association was observed between breast cancer frequency and ethnicity, mortality from breast cancer was shown to be slightly higher among Asian-Indian compared to African-descent populations in Trinidad (OR 1.2, 95% CI 1.1–1.4) and Guyana (OR 1.3, 95% CI 1.0–1.6).

**Conclusion:**

Study quantity, quality, and variability in outcomes and reporting limited the synthesis of evidence on the role of social determinants on breast cancer in the Caribbean. This report represents important current evidence on the region, and can guide future research priorities for better describing and understanding of Caribbean breast cancer inequalities.

**Electronic supplementary material:**

The online version of this article (doi:10.1186/s12939-017-0540-z) contains supplementary material, which is available to authorized users.

## Background

Among females in the Caribbean, breast cancer was the leading cause of cancer deaths, and accounted for 1.4 million disability adjusted life years (DALYs) in 2013 [1–3]. Age-standardized breast cancer mortality rates in the Caribbean have shown a 37% increase to 20.6 per 100,000 since 1990; this is in contrast to the decrease seen among many industrialised countries [1, 2].

Despite this high regional burden, little is known about the social distribution of breast cancer incidence and outcomes within the Caribbean. Internationally, social inequalities in breast cancer burden and outcomes are evident, such as by race and education [4–8]. Examining whether there are differences among populations groups, and determining their basis, can guide policy towards improving outcomes.

In 2007, the Port of Spain Declaration was affirmed by Caribbean Community (CARICOM) Heads of Government, aimed at the prevention and control of non-communicable diseases (NCDs), and there is an ongoing progress evaluation of political responses to this commitment [9, 10]. The World Health Organization (WHO) Commission on the Social Determinants of Health (CSDH) has highlighted the role of health research in understanding health inequalities and inequities, and through the 2011 Rio Political Declaration, countries have committed to monitoring, understanding and addressing health inequities [11, 12]. These agreements have set the scene for efforts to understand the social drivers of chronic disease, including cancers.

To date, there has been no published systematic review of research evidence on the social determinants of breast cancer among Caribbean populations. This systematic review is guided by the analytical framework to examine social determinants of disease by the WHO CSDH [13]. This review uses a simplified version of the framework to answer the primary research question: what is the distribution, by known social determinants of health, of the risk factors, frequency, and adverse outcomes of breast cancer among female populations living in the Caribbean?

## Methods

Full details of the review methodology are available in the study protocol (see Additional File 1). The protocol was guided by a previous systematic review of social determinants of diabetes [14] and an initial scoping review of the social determinants of breast cancer.

### Eligibility criteria

Observational studies were sought that reported relationships between a social determinant and known risk factors for breast cancer (alcohol intake, overweight/obesity, infrequent breastfeeding, physical inactivity, dietary sugar, ionizing radiation, late age at first pregnancy, and low parity), disease frequency (incidence or prevalence), or disease outcomes (cancer stage at diagnosis, cancer grade at diagnosis, recurrence, survival, mortality). Articles written in the dominant Caribbean languages (English, Spanish, French, and Dutch) were sought from 32 Caribbean territories. Included studies drew upon samples from either the general population or from healthcare facility catchments. No age restrictions were used in determining study eligibility. Sample sizes ≤50 were excluded as unlikely to be representative of underlying populations. Risk factors were identified using three compendiums of evidence-based information: The Global Burden of Disease Consortium, UpToDate, and Cancer Epidemiology and Control [15–17]. Articles presenting risk factor data from a sample of combined genders or males only were excluded so as to more accurately represent the risk factor profile in females. The selection of social determinants was guided by the extension of the PRISMA statement for the transparent reporting of systematic reviews and meta-analyses with a focus on health equity, which recommends the “PROGRESS-Plus” checklist: place of residence, race or ethnicity (alternatively culture or language), occupation, gender, religion, education, socio-economic position (SEP), and social capital [18]. Age was not examined as a social determinant for overweight/obesity and breast cancer frequency and outcomes due to its biological associations with these variables. Reports published between January 2004 and December 2014 were considered for inclusion. This 10-year period was selected as relevant to the current situation and able to inform policy response as it is taking place within the context of a major review of regional and national policy responses in the Caribbean to NCDs [10].

### Search strategy, study selection, data extraction

The databases searched were: MEDLINE (via Pubmed); EMBASE (via Ovid); SciELO; CINAHL (via EBSCO); CUMED, LILACS, and IBECS (via WHO Virtual Health Library) [19–23]. The final search was conducted in February 2015. The search strategies are detailed in a supplementary file (See Additional File 2). Search results were maintained in Endnote reference management software [24].

Study selection and data abstraction were undertaken in duplicate by two independent reviewers (CB, SH); any inconsistencies were resolved by a third reviewer (NS-G). Study selection was conducted in two stages. First, titles and abstracts were screened to identify potentially relevant articles; second, full-text screening of potentially relevant articles identified articles for inclusion in the review. If inadequate information was available for decision-making in the first stage, the article automatically progressed to full-text review. In addition to those not meeting the inclusion criteria, 10 articles were either inaccessible or awaiting publication [25–34]. With guidance by the STROBE statement on strengthening the reporting of observational studies in epidemiology and the PRISMA-Equity statement [35, 36], an electronic data abstraction form was created in the REDCap database (see Additional file 1) [37].

### Risk of bias assessment

Risk of bias was assessed using a tool adapted from STROBE and Cochrane ACROBAT-NRSi guidelines (see Additional file 1) [35, 38]. Bias was assessed at the relationship level across 5 domains: confounding (was control for known and potential confounders adequate?); participant selection (is the sample representative of the target population?); missing data (is the data reasonably complete?); outcome measurement (is a social determinant/risk factor/disease endpoint appropriately measured?); selective reporting (is a relationship selectively reported?). Articles were classified as having serious, moderate, low, or unclear risk of bias. Two reviewers (CB, NS-G) made an independent judgement on the overall risk of bias of each included article, considering each domain as equally important and also the direction and magnitude of the bias from each domain. Discrepancies were discussed by the two reviewers to achieve consensus.

### Synthesis of results

The review was planned as a narrative synthesis with supplementary meta-analysis if possible. Key study details were presented, followed by a description of associations between a social determinant and either a risk factor, a measure of disease frequency, or a measure of disease outcomes. The number and type of inequality relationships were summarised in an ‘evidence gap map’ – a visual tool to highlight the current evidence on the known social determinants of breast cancer in the Caribbean and a guide for focusing future research [39]. Meta-analysis of quantitative evidence was planned for inequality relationships reported by ≥2 studies with low to moderate heterogeneity and classified as having a low or moderate risk of bias [38]. Meta-analysis was not performed because of lack of sufficient evidence (number and quality) for each domain of social indicators.

## Results

### Summary of included studies

**Fig. 1:**
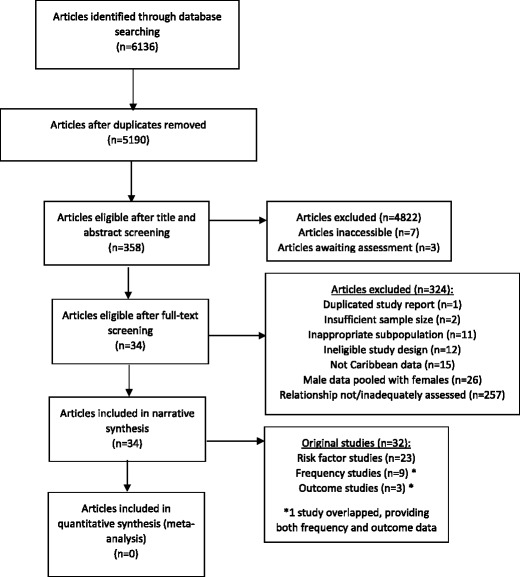
Flowchart of search strategy and article selection

Thirty-four articles from 32 original studies were included from 5,190 screened citations (Fig. 1). Of these 34 articles, 23 reported on breast cancer risk factors, 9 reported on breast cancer frequency, and 3 reported on breast cancer outcomes (1 article examined both breast cancer frequency and outcomes); 10 social determinants were examined (Table 1).

**Table 1:** Characteristics of 34 articles describing the social distribution of breast cancer in Caribbean women [40–70, 72, 73]

Study-level characteristics							Inequality relationships reported			Main Findings
Article (*n* = 34)	Study design	Sample size	Age range	Study-base	Country	Proxies used	Risk Factor	Frequency	Outcome	
Agyemang, 2009 [46]	Cross-sectional	855	12 to 17	School	Suriname		Ethnicity^O^	/	/	Mean BMIs across ethnicity: Hindu (19.5 ± 4.0), Creole (20.8 ± 3.8), Javanese (19.3 ± 3.0), Maroon (21.3 ± 4.1), mixed (20.3 ± 3.5). *p* = 0.02. Maroon girls had a higher BMI than Hindustani girls (*p* = 0.03) and Javanese (*p* < 0.01) girls.
							Ethnicity^PI^			Proportions of girls who exercise ≥5–7 days per week across ethnicities: Hindu (7.4%), Creole (6.0%), Javanese (4.4%), Maroon (4.0%), mixed (8.2%). *p* = 0.74
Alvarez, 2009 [63]	Registry-based	/	(all)	Population	Cuba		/	Residence^I^	/	Moderately higher risk for breast cancer (CAR smoothed RR of 1.21–1.26) observed in La Cuidad de Habana and two nighboring districts of Matanzas relative to the national Cuban average, but there were no significant rural/urban distictions among these and other municipalities examined.
Block, 2012 [40] (a)	Cross-sectional	2,017	18 to 104	Population	Grenada		Age^Alc^	/	/	Proportion of women who consume 1–2 drinks/day or 1–7 drinks/week across age groups: <35 (3.2%), 35–44 (4.9%), 45–54 (6.5%), 55–64 (7.6%), >64 (2.2%). *p* = 0.93
							Age^PI^			Proportion women who walk/bike continuously for >10 min/day across age groups: <35 (79.5%), 35–44 (81.1%), 45–54 (80.1%), 55–64 (7.6%), >64 (2.2%). *p* = <0.001. Proportion of women who spend leisure time sedentary for >10 min/day across age groups: <35 (78.1%), 35–44 (79.9%), 45–54 (82.9%), 55–64 (83.8%), >64 (83.5%). *p* = 0.53.
Blum, 2004 [41] (a)	Cross-sectional	15,695	10 to 18	School	Antigua, Bahamas, Barbados, British Virgin Islands, Dominica, Guyana, Jamaica, St. Lucia	Religious attendance	Religion^Alc^	/	/	For girls attending religious service within the past 3 months, the odds for alcohol use weekly or daily is is 0.50 (*p* < 0.001).
Brathwaite, 2011 [47] (a)	Cross-sectional	6,947	21 to 60	Population	Bahamas	Education – (in addition to individual) maternal education, paternal education; Income – household expenditure	Education^O,O,O^	/	/	Proportions and CI of obesity across levels of maternal education: primary school or less (44.6%, 37.9–51.5), high school (29.3%, 23.7–35.7), technical or vocational (43.0%, 14.1–77.6), college/university (20.1%, 10.8–34.5). *p* = 0.002. Proportions and CI of obesity across levels of paternal education: primary school or less (41.4%, 34.4–48.7), high school (31.7%, 26.3–37.6), technical or vocational (18.7%, 4.9–50.5), college/university (21.7%, 12.3–35.5). *p* = 0.021. Proportions and CI of obesity across levels of individual education: primary school or less (36.7%, 25.9–49.0), high school (43.9%, 38.6–49.4), technical or vocational (26.6%, 13.3–46.2), college/university (24.6%, 18.6–31.9). *p* = 0.0001. Logistic regression shows participant education to predicts obesity (OR 0.706, 95% CI 0.586–0.850, *p* = <0.00001)
							Residence^O^			Proportions and CI of obesity by residence type: nonurban (43.8%, 38.0–49.8), urban (37.0%, 32.4, 41.9).*p* = 0.080.
							Income^O^			Proportions and CI of obesity are as follows across income levels: 1/poorest (40.1%, 31.2–49.7), 2 (49.5%, 39.6–59.5), 3 (42.4%, 34.3–51.0), 4 (32.3%, 25.8–39.5), 5/wealthiest (29.9%, 23.5–37.1). *p* = 0.006.
							Social household structure^O^			Proportions and CI of obesity across household heading: non-female headed household (37.0%, 32.1–42.3), female headed household (38.6%, 32.7–44.9). *p* = 0.678.
Bryan, 2012 [48]	Cross-sectional	801	18+	Region/community	Jamaica	Health insurance status	Income^O^	/	/	BMI mean ranks across health insurance status: has health insurance (452.18), does not have health insurance (383.32), does not know (277.80). “Body mass index was higher for those with health insurance”. (p value not given)
Chatman, 2004 [60]	Cross-sectional	599	14 to 45	Health facility	Jamaica		Age^Bf^	/	/	Proportions of breastfeeding (exclusive, nonexclusive): <20 (14.3%, 12.5%), 20–29 (52.6%, 55.8%), >29 (33.1%, 31.7%). *p* = 0.8. Regression results not shown – but age stated to not be a significant predictor of breastfeeding exclusivity.
							Education^Bf^			Proportions of breastfeeding (exclusive, nonexclusive): no education (0.75%, 0.2%), primary education (27.8%, 22.2%), beyond primary education (71.4%, 77.4%), missing information (0%, 0.2%). *p* = 0.4. Regression results not shown – but education stated to not be a significant predictor of breastfeeding exclusivity.
							Income^Bf^			Proportions of breastfeeding (exclusive, nonexclusive) by main source of income: mother (11.3%, 10.3%), father (53.4%, 36.1%), other (35.3%, 53.6%). *p* = 0.0005. Regression results not shown – but source of income stated not to be a significant predictor of breastfeeding exclusivity.
							Marital status^Bf^			Proportions of breastfeeding (exclusive, nonexclusive): single (39.1%, 40.0%), married (21.1%, 16.9%), common law (39.9%, 43.1%). *p* = 0.8. Regression results not shown – but marital status stated to not be a significant predictor of breastfeeding exclusivity.
							Occupation^Bf^			Proportions of breastfeeding (exclusive, nonexclusive) across maternal job status: employed (21.1%, 31.0%), unemployed (79.0%, 68.8%), missing (0%, 0.2%). *p* = 0.07. Proportions of breastfeeding (exclusive, nonexclusive) across paternal job status: employed (88.7%, 92.3%), unemployed (9.8%, 6.4%), not sure (1.5%, 1.3%). *p* = 0.4. Regression found that when the father was the main financial source for the family as compared families with the mother was the main source of income, the likelihood of exclusive breastfeeding was doubled (adjusted OR 2.03; 95% CI 1.4–3.0).
Dubois, 2011 [49] (a)	Cross-sectional	1,674	10 to 11	School	Jamaica	SEP – household crowding, geographical index	SEP^O^	/	/	Proportion of overweight/obesity across SES levels: low (8.3%), medium (14.9%), high (14.2%). *p* = ≥0.05. Regression for overweight/obesity across SES levels: medium (OR 1.87, 95% CI 1.0–3.4), high (OR 1.74, 95% CI 0.9-3.3) (ref: low).
							Social household structure^O^			Proportion of overweight/obesity across family structure: two-parent family (13.8%), blended family (13.5%), single-parent (10.8%). *p* = ≥0.05). Regression for overweight/obesity across family structure: blended family (OR 1.0, 95% CI 0.6–1.6), single-parent (OR 0.79, 95% CI 0.4–1.3) (ref: two-parent family).
Ferguson, 2011 [50] (a)	Cross-sectional	839	18 to 20	Population	Jamaica	Education – parental education; Occupation – head of household occupation	Education^O^	/	/	Prevalence of elevated waist circumference according to parental education: tertiary (12.50%), secondary (14.08%), primary/all age (28.43%), don’t know (18.18%). *p* = 0.002 for association; *p* = 0.002 for trend. Regression for central obesity accross to parental education: secondary (OR 1.72, 95% CI 0.74–4.01, *p* = 0.205), primary/all age (OR 6.14, 95% CI 2.05-18.40, *p* = 0.001), don’t know (OR 4.61, 95% CI 1.47–14.39, *p* = 0.009) (reference: tertiary).
							Occupation^O^			Prevalence of elevated waist circumference according to head of household occupation status: highly skilled (12.43%), skilled (13.55%), semi/unskilled (21.84%), other (22.81%). *p* = 0.013 for association; *p* = 0.009 for trend. Regression for central obesity accross parental occupation: skilled (OR 2.55, 95% CI 0.99–6.57, *p* = 0.054), semi/unskilled (OR 3.37, 95% CI 1.22–9.29, *p* = 0.019), other (OR 4.67, 95% CI 1.17–18.55, *p* = 0.029) (ref: highly skilled).
Grievink, 2004 [51]	Cross-sectional	2,025	18+	Population	Bonaire, St. Eustatius, Saba		Education^O^	/	/	Proportion and regression for obesity across education level: low (36.8%, reference), intermediate (36.7%, OR 0.9, 95% CI 0.6–1.3), high (27.2%, OR 0.6, 95% CI 0.4-0.9). Proportion and regression for high waist circumference across education level: low (69.4%, reference), intermediate (50.8%, OR 0.7, 95% CI 0.5–0.9), high (44.0%, OR 0.5, 95% CI 0.3–0.7). Proportion and regression for high waist to hip ratio across eduation level: low (78.4%, reference), intermediate (65.3%, OR 1.0, 95% CI 0.7–1.6), high (55.1%, OR 0.6, 95% CI 0.4–0.9).
							Income^O^			Proportion and regression for obesity across income level: <825 USD (34.9%, reference), 825–1650 USD (33.9%, OR 1.0, 95% CI 0.7–1.4), >1650 (33.3%, OR 0.9, 95% CI 0.6–1.4). Proportion and regression for high waist circumference across income level: <825 USD (58.3%, reference), 825–1650 USD (54.3%, OR 1.0, 95% CI 0.7–1.5), >1650 (51.2%, OR 1.0, 95% CI 0.7–1.4). Proportion and regression high waist to hip ratio across income level: <825 USD (70.7%, reference), 825–1650 USD (65.0%, OR 1.0, 95% CI 0.7–1.4), >1650 (56.8%, OR 0.7, 95% CI 0.5–1.1).
							Occupation^O^			Proportion and regression for obesity across occupation level: low (36.8%, reference), intermediate (36.7%, OR 0.9, 95% CI 0.6–1.3), high (27.9%, OR 0.7, 95% CI 0.5–0.9). Proportion and regression for high waist circumference across occupation level: low (57.1%, reference), intermediate (54.8%, OR 1.0, 95% CI 0.7–1.4), high (50.8%, OR 0.8, 95% CI 0.6–1.1). Proportion and regression for high waist to hip ratio across occupation level: low (67.6%, reference), intermediate (66.4%, OR 1.0, 95% CI 0.7–1.5), high (63.8%, OR 0.8, 95% CI 0.6–1.2).
Hernández, 2013 [64]	Registry-based	/	(all)	Region/community	Cuba		/	Residence^I^	/	There existed spacial clustering (RR 1.63, *p* = 0.015) and spacial-time clustering (RR 1.91, *p* = 0.016) of breast cancer incidence in: Encrucijada, Camajuani, Caibarien, Santa Clara, but not in the other municipalities. But there were no significant rural/urban distictions among these and other municipalities examined.
Ichinohe, 2005 [52]	Cross-sectional	1,935	/	Population	Jamaica		Education^O^	/	/	Regression for education as a predictor of BMI: β -0.560, CI −0.795–0.325, *p* = 0.000. There is a lower prevelance of obesity in those with more education.
							Marital status^O^			Regression for marital status as a predictor of BMI: β -0.168, CI −0.329–0.007, *p* = 0.041. There is a lower prevalence of obesity in married persons.
Joseph, 2014 [65]	Cross-sectional	2,582	/	Health facility	Trinidad & Tobago		/	Ethnicity^C^	/	Regression for incident breast cancer cases: white (crude OR 1.22, 95% CI 0.36–4.06; adjusted OR 1.42, 95% CI 0.4–5.0), East Indian (crude OR 0.99, 95% CI 0.54–1.82; adjusted OR 0.98, 95% CI 0.47–2.04), mixed (crude OR 0.83, 95% CI 0.5–1.37; adjusted OR 0.79, 95% CI 0.43–1.44), Asian and other (crude OR 0.71, 95% CI 0.09–5.35; adjusted OR 0.76, 95% CI 0.44–1.20), missing (crude OR 0.83, 95% CI 0.54–1.28; adjusted OR 0.73, 95% CI 0.44–1.20) (ref: African ancestry).
								Marital status^C^		Proportions (#) of incident breast cases: single/separated/widowed/divorced (62), married/common law (66), missing (3). Regression for incident breast cancer cases: married/common law (crude OR 0.82, 95% CI 0.58–1.17), missing (crude OR 0.92, 95% CI 0.28–3.02) (ref: single/separated/widowed/divorced).
Kim, 2007 [42] (a)	Cross-sectional	3,408	60+	Region/community	Barbados, Cuba		Age^Alc^	/	/	Barbados: Proportions of older adults who consumed alcohol ≥4 days/week across age group: 60–65 (3.2%), 66–70 (2.0%), 71–75 (2.2%), 76–80 (2.5%), >80 (3.2%). Cuba: Proportions of older adults who consumed alcohol ≥4 days/week across age group: 60–65 (1.8%), 66–70 (0.6%), 71–75 (0.4%), 76–80 (1.6%), >80 (0.9%).
							Education^Alc^			Barbados: Proportions of older adults who consumed alcohol ≥4 days/week, across years of education: none (unreliable data), 1–6 (1.1%), 7–12 (8.1%), >12 (11.2%). Cuba: Proportions of older adults who consumed alcohol ≥4 days/week, across years of education: none (0.0%), 1–6 (1.1%), 7–12 (1.3%), >12 (1.5%).
							Marital status^Alc^			Barbados: Proportions of older adults who consumed alcohol ≥4 days/week: union (4.8%), other (2.1%). Cuba: Proportions of older adults who consumed alcohol ≥4 days/week: union (1.2%), other (1.1%).
							Residence^Alc^			Proportions of older adults who consumed alcohol ≥4 days/week: Barbados (2.7%), Cuba (1.1%)
Laborde, 2013 [53] (a)	Cross-sectional	6025	(all)	Population	Puerto Rico		Education^O^	/	/	Regression for overweight: college (OR 1.060, 95% CI 0.904–1.243, *p* = 0.472) (ref: no college). Regression for class 1 obese: college (OR 0.819, 95% CI 0.672–0.999, *p* = 0.048) (ref: no college). Regression for class 2/3 obese: college (OR 0.586, 95% CI 0.469–0.734, *p* = 0.000) (ref: no college).
							Income^O^			Regression for overweight across income bracket: $15000–24999 (OR 1.143, 95% CI 0.962–1.358, *p* = 0.130), $25000–49000 (OR 1.148, 95% CI 0.926–1.422, *p* = 0.209), >$49000 (OR 0.887, 95% CI 0.651–1.209, *p* = 0.447) (ref:<$15000). Regression for class 1 obesity: $15000–24999 (OR 1.131, 95% CI 0.914–1.400, *p* = 0.259), $25000–49000 (OR 1.064, 95% CI 0.810–1.398, *p* = 0.657), >$49000 (OR 0.777, 95% CI 0.510–1.183, *p* = 0.239) (ref:<$15000). Regression for class 2/3 obesity: $15000–24999 (OR 0.662, 95% CI 0.519–0.846, *p* = 0.001), $25000–49000 (OR 0.540, 95% CI 0.385–0.757, *p* = 0.000), >$49000 (OR 0.255, 95% CI 0.130–0.499, *p* = 0.000) (ref:<$15000).
							Marital status^O^			Regression for overweight: married (OR 1.029, 95% CI 0.894–1.185, *p* = 0.690) (ref: not married). Regression for class 1 obesity: married (OR 1.210, 95% CI 1.016–1.442, *p* = 0.032) (ref: not married). Regression for class 2/3 obesity: married (OR 0.969, 95% CI 0.794–1.181, *p* = 0.752) (ref: not married).
Latimer, 2004 [43]	Cross-sectional	972	11 to 19	School	Puerto Rico		Age^Alc^	/	/	Proportions of lifetime, 12-month, and 3-month alcohol use: middle school age groups (58.3%, 42.1%, 31.6%), high school age groups (77.0%, 57.3%, 31.6%).
Mendez, 2004 [54] (a)	Cross-sectional	2,096	25 to 74	Population	Jamaica		Income^O^	/	/	Proportions and regression for overweight across monthly income: <$1000 (30.4%, reference), $1000–3000 (32.7%, OR 0.96, 95% CI 0.65–1.42), $3001–6000 (31.7%, OR 1.61, 95% CI 1.04–2.48), >$6000 (36.9%, OR 1.70, 95% CI 0.97–2.98). Proportion and regression for obesity across monthly income: <1000 (32.5%, reference), 1000–3000 (26.1%, OR 0.75, 95% CI 0.50-1.13), 3001–6000 (41.8%, OR 1.83, 95% CI 1.19–2.80), >6000 (34.4%, OR 1.66, 0.95–2.92). Multivariate ORs comparing prevalence in women above vs below the poverty line were significant for overweight and obesity.
Morales, 2013 [66]	Case–control	1,126	21+	Population	Puerto Rico		/	Education^C^	/	Regression for breast cancer: grades 1–8 (crude OR 5.77, 95% CI 2.9-11.7; adjusted OR 3.38, 95% CI 1.5-5.7; *p* = 0.003), grades 9–12 (crude OR 1.72, 95% CI 1.3–2.2; adjusted OR 1.33, 95% CI 0.9–1.9; *p* = 0.086) (ref: associate or higher degree).
								Marital status^C^		Regression for breast cancer: divorced (crude OR 3.59, 95% CI 2.1–5.8; adjusted OR 2.57, 95% CI 1.4–4.4; *p* = 0.002), single (crude OR 2.11, 95% CI 1.2–3.6; adjusted OR 1.36, 95% CI 0.7–2.6; *p* = 0.421), widow (crude OR 2.74, 95% CI 1.5–5.0; adjusted OR 2.08, 95% CI 1.1–4.0; *p* = 0.039) (ref: married).
Nam, 2012 [55] (a)	Cross-sectional	5,786	65+	Region/community	Barbados, Cuba		Education^O^	/	/	Barbados: Mean years of education: low waist circumference (5.1 ± 0.2), high waist circumference (5.2 ± 0.2). *p* > 0.01. Cuba: Mean years of education: low waist circumference (6.2 ± 0.2), high waist circumference (6.5 ± 0.2), *p* > 0.01.
							Marital status^O^			Barbados: Proportion of married females: low waist circumference (24.6%), high waist circumference (22.6%). *p* > 0.01. Cuba: Proportion of married females: low waist circumference (11.1%), high waist circumference (19.5%). *p* < 0.001.
							Residence^O^			Proportion of women with high waist circumference: Barbados (63%), Cuba (48.5%).
Nemesure, 2009 [67] (a)	Case control	722	21+	Population	Barbados		/	Education^C^	/	Mean years of education: breast cancer cases (12.1 ± 3.8,), controls (11.7 ± 3.3). *p* = 0.13
								Marital status^C^		Proportion of marital status types (breast cancer cases, controls): single and never married (30.2%, 35.7%), married or living together (42.3%, 41.0%), separated or divorced (14.9%, 11.9%), widowed (12.6%, 11.4%). *p* = 0.46.
								Occupation^C^		Proportion of occupations (breast cancer cases, controls): housewife/homemaker (11.3%, 7.1%), professor/administrative/managerial (19.4%, 13.2%), other (69.4%, 79.7%). *p* = 0.01. Regression for breast cancer: professional occupation (OR 1.36, 95% CI 0.83–2.24), housewife/homemaker (OR 1.58, 95% CI 0.86–2.89), (ref: other).
Ohene, 2005 [44] (a)	Cross-sectional	15,695	10 to 18	School	Antigua, Bahamas, Barbados, British Virgin Islands, Dominica, Grenada, Guyana, Jamaica, St. Lucia		Age^Alc^	/	/	Proportions of alcohol use within past 12 months, across age group: 10–12 (3.1%) 13–15 (7.3%), 16–18 (11.1%)
Pérez-Ríos, 2008 [61] (a)	Cross-sectional	1,695	15 to 49	Population	Puerto Rico		Age^Bf^	/	/	Proportion of women initiating breastfeeding across age group: 15–24 (61.3%), 25–34 (67.7%), 35–49 (61.4%). *p* = 0.024. Regression for breastfeeding initiation: 25–34 (crude OR 0.76, 95% CI 0.60–0.95; adjusted OR 1.04, 95% CI 0.81–1.35), 35–49 (crude OR 1.00, 95% CI 0.74–1.34; adjusted OR 1.39, 95% CI 1.00–1.95) (ref: 15–24).
							Education^Bf^			Proportion of women initiating breastfeeding across education level: 0–8 school years (49.5%), 9–11 school years (55.3%), high-school diploma (62.9%), associate degree/some university without diploma (70.0%), baccalaureate/postgraduate (81.2%). *p* = 0.0001. Regression for breastfeeding initiation: 9–11 school years (crude OR 0.79, 95% CI 0.55–0.08; adjusted OR 0.88, 95% CI 0.60–1.29), high-school diploma (crude OR 0.58, 95% CI 0.42–0.08; adjusted OR 0.67, 95% CI 0.47–0.94), associate degree/some university without diploma (crude OR 0.42, 95% CI 0.30–0.59; adjusted OR 0.49, 95% CI 0.34–0.72), baccalaureate/postgraduate (crude OR 0.23, 95% CI 0.15–0.34; adjusted OR 0.29, 95% CI 0.17–0.45).
							Marital status^Bf^			Proportion of women initiating breastfeeding: married (70.2%), living together (54.5%), without a partner (57.6%). *p* = 0.0001. Regression for breastfeeding initiation: living together (crude OR 1.96, 95% CI 1.53–2.52; adjusted OR 1.55, 95% CI 1.18–2.05), without a partner (crude OR 1.73, 95% CI 1.33–2.26; adjusted OR 1.45, 95% CI 1.09–1.92) (ref: married).
							Occupation^Bf^			Proportion of women initiating breastfeeding: employed (71.9%), unemployed (61.0%,). *p* = 0.0001. Regression for breastfeeding initiation: employed (crude OR 1.63, 95% CI 1.31–2.03; adjusted OR 1.15, 95% CI 0.89–1.48) (ref: unemployed).
Rivera-Lugo, 2007 [62]	Cross-sectional	200	22+	Health facility	Puerto Rico		Age^Bf^	/	/	Results not stated because simple logistic regression showed a *p*= > 0.10 for exclusive postpartum breastfeeding.
							Education^Bf^			Regression for exclusive postpartum breastfeeding: high school or less (OR 0.354, 95% CI 0.046–2.736, *p* = 0.320), vocational/associate degree (OR 0.649, 95% CI 0.168–2.511, *p* = 0.531), some college level (OR 0.807, 95% CI 0.190–3.435, *p* = 0.772), bachelor degree (OR 1.145, 95% CI 0.384–3.416, *p* = 0.808) (ref: masters/doctorate).
							Income^Bf^			Regression for exclusive breastfeeding: $0–2000 (OR 0.301, CI 0.082–1.112, *p* = 0.072), $2001–3000 (OR 0.460, CI 0.140–1.514, *p* = 0.201), $3001–$4000 (OR 0.317, CI 0.101–0.994, *p* = 0.049) (ref: >$4000) (reference).
							Marital status^Bf^			Results not stated because simple logistic regression showed a *p*= > 0.10 for exclusive postpartum breastfeeding.
Santana, 2011 [72]	Registry-based	1,819	(all)	Region/community	Cuba		/	/	Residence	Number of deaths and crude mortality rates (per 100,000) respectively of prostate cancer across municipality: Contramaestre (6, 11.7), Mella (5, 28.9), San Luis (7, 15.9), II Frente (2, 10.3), Songo-La Maya (10, 21.6), Santiago (72, 28.5), Palma (8, 13.0), III Frente (3, 21.3), Guamá (2, 11.9). Weak preponderance of prosatate cancer in more urban areas (no significance testing done).
Shirley, 2010 [68]	Registry-based	772	21 to 96	Population	Jamaica		/	Residence^C^	/	Proportion of incident breast cancer cases by parish: Kingston & St. Andrew (34.7%), Manchester (22.9%), St. Catherine (13.9%), St. Ann (7.3%), St. Mary (5.1%), St. Thomas (4.4%), St. James (3.9%), Portland (3.2%), St. Elizabeth (2.5%), Clarendon (1.9%). No urban/rural trend found (no significance testing done)
Sinnapah, 2009 [56]	Cross-sectional	780	10 to 18	School	Guadeloupe		Ethnicity^O,PI^	/	/	ETHNICITY - Means of daily duration of leisure-time physical activity (“LTPA”) (hours/day): Asian-Indians (1.25 ± 1.19), other (1.51 ± 1.29). Means of absolute time spent in activities (light, moderate, vigorous): Asian-Indian (2.9 ± 3.8, 3.2 ± 4.2, 2.5 ± 3.9), other (2.3 ± 4.4, 4.2 ± −5.0, 3.9 ± 5.1). Means of average intensity of LTPA (MET): Asian-Indian (4.5 ± 1.7), other (5.0 ± 1.9). Means of maximal intensity of LTPA (MET): Asian-Indian (7.1 ± 2.3), other (7.7 ± 2.7). OVERWEIGHT/OBESITY - Mean BMI: Asian-Indian (18.8 ± 3.0), other (20.2 ± 3.7). *p* < 0.05.
Sinnapah, 2009 [57]	Cross-sectional	122	17 to 66	Health facility	Guadeloupe		Ethnicity^O,PI^	/	/	ETHNICITY -Mean physical activity levels: Asian-Indian (1.62 ± 0.22), other (1.74 ± 0.34). *p* = <0.05. OVERWEIGHT/OBESITY - Means of BMI: Asian-Indians (24.4 ± 4.0), others (24.4 ± 4.3). *p* > 0.05.
Sinnapah, 2009 [58]	Cross-sectional	720	11 to 17	School	Guadeloupe		Ethnicity^O^	/	/	Results are stratified by age groups - <14 and >14. Mean BMI (<14 and >14): Asian Indian (19.0 + 3.5, 21.1 + 5.3), other (20.3 + 4.0, 21.4 + 4.0). *p* > 0.05. Mean waist circumference: Asian Indian (65.5+/−8.8, 68.1+/−8.7), other (68.5+/−8.7, 70.2+/−8.9). *p* = <0.05. Mean waist to hip ratio: Asian Indian (0.75 + 0.04, 0.74 + 0.06), other (0.76 + 0.05, 0.73 + 0.04). *p* < 0.05. Mean % body fat: Asian Indian (25.2 + 5.7, 26.6 + 5.2), other (24.3 + 5.5, 25.1 + 5.9). *p* < 0.05. Proportions of obesity (all ages): Asian Indian (2.2%), other (7.2%). No p-value given.
Taioli, 2012 [73]	Registry-based	3,710	all	Population	Trinidad & Tobago, Guyana		/	/	Ethnicity	Trinidad: Regression for breast cancer mortality across ethnicity: white (HR 1.3, 95% CI 0.8–1.9), Indian (HR 1.2, 95% CI 1.1–1.4), other/unknown (HR 1.3, 95% CI 1.1–1.5) (ref: black). Guyana: Regression for breast cancer mortality across ethnicity: white (HR 1.1, 95% CI 0.4–2.6), Indian (HR 1.3, 95% CI 1.0–1.6), other/unknown (HR 1.0, 95% CI 0.7–1.5).
Torres, 2007 [69]	Cross-sectional	/	25 to 50	Population	Cuba		/	Residence^I^	/	Means (range) of incidence rates per 100,000 are as follows - Pinar del Rio, Havana, Cienfuegos,Villa Clara, Ciego de Avila (≤20.7); Sancti Spiritus, Matanzas, Isla de Juventud (20.8-24.9); Camaguey, Holguin, Granma (25.0-36.8); Santiago de Cuba, Guantanamo, Las Tunas (≥36.9). No urban/rural differences.
Torres-Cintrón, 2010	Registry-based	/	(all)	Population	Puerto Rico		/	Residence^I^	Residence	INCIDENCE - Standardized incidence (per 100,000), rate ratios and CI across regions of Puerto Rico: Northwest (70.8, 0.99, 0.91–1.08), North (64.3, 0.90, 0.84–0.97), Central (72.4, 1.01, 0.95–1.07), East (64.7, 0.90, 0.80–1.02), Northeast (77.1, 1.08, 1.03–1.13), Southeast (58.5, 0.82, 0.76–0.88), South (64.0, 0.89, 0.84–0.96), Southwest (70.4, 0.98, 0.90–1.07). Figures for the North, Northeast, Southeast, and South are significantly different from overall Puerto Rico (*p* < 0.05), but there were no significant rural/urban distinctions between these and other municipalities examined. MORTALITY - Standardized mortality (per 100,000), rate ratios and CI across regions of Puerto Rico: Northwest (13.3, 0.81, 0.66–0.99), North (13.8, 0.85, 0.72–0.99), Central (172., 1.05, 0.93–1.19), East (20.4, 1.25, 0.99–1.56), Northeast (19.1, 1.17, 1.06–1.29), Southeast (15.2, 0.93, 0.81–1.06), South (14.7, 0.90, 0.78–1.03)), Southwest (15.8, 0.97, 0.81–1.15) (ref: Puerto Rico). Figures for the North, Northwest, and Northeast are significantly different from overall Puerto Rico (*p* < 0.05), but there were no significant rural/urban distinctions between these and other municipalities examined.
Tull, 2005 [59]	Cross-sectional	893	20+	Regional/community	US Virgin Islands (St. Croix only)		Ethnicity^O^	/	/	Proportions and CI of overweight: Hispanic white (30.7%, 8.7–52.7), Hispanic black (35.6%, 23.0–48.2), nonhispanic black immigrant (33.9%, 26.3–41.5), nonhispanic black USVI-born (26.75, 16.0–37.4). *p* > 0.05. Proportions and CI obesity: Hispanic white (43.5%, 26.5–61.4), Hispanic black (35.6%, 23.0–48.2), nonhispanic black immigrant (44.3%, 37.9–50.7), nonhispanic black USVI-born (38.8%, 29.9–47.7).
van Leeuwaarde, 2011 [70]	Registry-based	/	(all)	Population	Suriname		/	Ethnicity^I^	/	Proportions and incidence rates (per 100,000 per year) of breast cancer: Creole (37.2%, 35.7), Maroons (1.9%, 2.2), Hindu (29.4%, 18.2), Javanese (17.9%, 20.8), Chinese (1.9%, not given), mixed (7.4%, 10.1), Dutch (1.4%, not given), other (2.9%, not given). Note these proportions also reflect the ethnography of the general Suriname population.
Varona, 2011 [45] (a)	Cross-sectional	22,851	15+	Population	Cuba	Income – perception of economic situation	Age^Alc^	/	/	Proportions and CI of females consuming alcohol in past 30 days across age group: 15–19 (11.4%, 8.9–13.9), 20–39 (14.6%, 13.2–15.9), 40–59 (9.3%, 8.0–10.5), >59 (2.7%, 1.8–3.6).
							Education^Alc^			Proportions and CI of females consuming alcohol in past 30 days: primary school (4.8%, 3.8–5.7), middle school (10.7%, 9.4–12.1), high school (13.9%, 12.4–15.4), university (13.2%, 10.8–15.7).
							Ethnicity^Alc^			Proportions and CI of females consuming alcohol in past 30 days: white (8.2%, 7.3–9.0), mestizo (14.7%, 12.9–16.5), black (14.9%, 12.3–17.6).
							Income^Alc^			Proportions and CI of females consuming alcohol in past 30 days: excellent (8.4%, 2.3–14.6), good (11.8%, 10.0–13.6), fair (10.2%, 9.2–11.1), poor (9.2%, 7.4–10.9), very poor (10.9%, 7.6–14.1).
							Marital status^Alc^			Proportions and CI of females consuming alcohol in past 30 days: unmarried (14.1%, 12.3–16.0), married or cohabiting (10.0%, 9.0–10.9), divorced or separated (12.0%, 9.9–14.0), widowed (2.5%, 1.4–3.7).
							Occupation^Alc^			Proportions and CI of females consuming alcohol in past 30 days: manager (18.8%, 13.8–23.7), administrator (14.7%, 10.5–18.8), upper-level technician (13.1%, 10.1–16.1), middle-level technician (12.6%, 10.0–16.1), labourer (14.0%, 10.5–17.2), service worker (16.0%, 13.2–18.8).

• (a) - Articles are components of larger studies: (Block, [40]) *- Grenada Heart Project* [100]*;* ((Blum, [41]), (Ohene, [44])) - *Caribbean Youth Health Survey* [101]; (Brathwaite, [47]) - *2001 Bahamas Living Conditions Survey* [102]*;* ((Nam, [55]), (Kim, [42])) – *[Health, Wellbeing and Aging]* [103]; (Dubois, [49]) - *Jamaica Youth Risk and Resiliency Behaviour Survey of 2007* [104]*;* (Ferguson, [50]) - *Jamaica Birth Cohort* [105]*;* (Laborde, [53]) - *Behavioral Risk Factor Surveillance System* [106]*;* (Mendez, [54]) - *International Collaborative Study on Hypertension in Blacks* [107]*;* (Nemesure, [67]) - *The Barbados National Cancer Study* [67]; (Pérez-Ríos, [61]) - *Puerto Rico Reproductive Health Survey* [108]*;* (Varona, [45]) – *2011 National Survey on Risk Factors and Chronic Diseases* [109]

• Social determinants listed under “Risk Factors” are designated as “Alc” for alcohol; “Bf” for limited breastfeeding; “O” for overweight/obesity; and “PI” for physical inactivity

• Social determinants listed under “Frequency” are designated as “I” for incidence and “C” for numbers of cases

• All social determinants listed under “Outcome” are examined by mortality

**Fig. 2:**
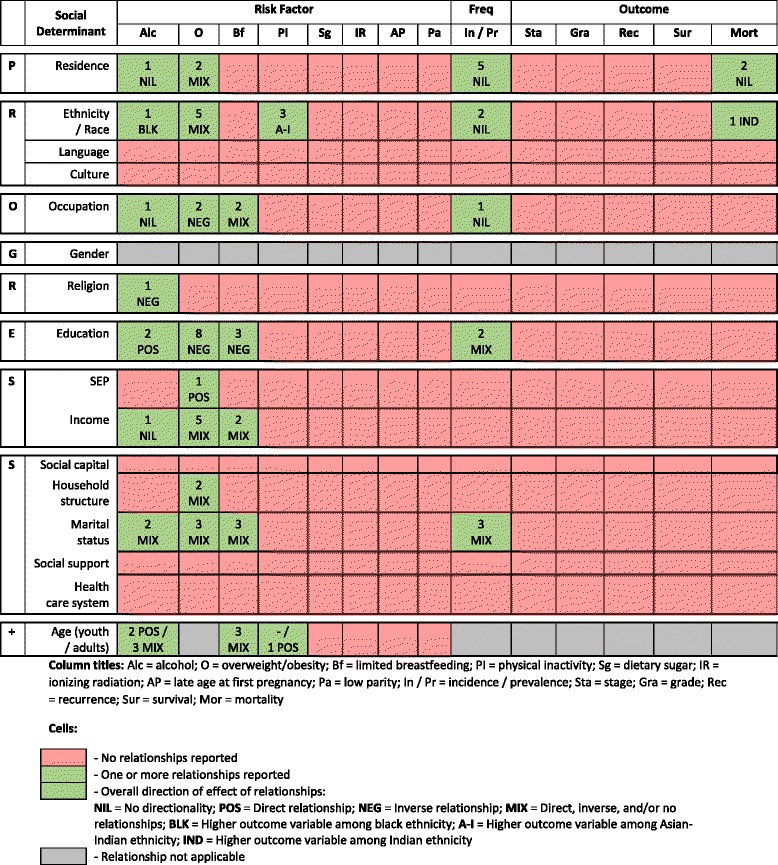
Summary of 75 inequality relationships from 34 articles between a social determinant and review endpoint [40–70, 72, 73]. Legend: Age and limited breastfeeding cells do not separate youth and adult samples as the studies have combined these age groups in their samples

Included articles reported on studies conducted in English-speaking (Antigua, Bahamas, Barbados, British Virgin Islands, Dominica, Grenada, Guyana, Jamaica, St. Lucia, Trinidad and Tobago, United States Virgin Islands); French-speaking (Guadeloupe); Dutch-speaking (Bonaire, St. Eustatius, Saba, Suriname); and Spanish-speaking (Cuba, Puerto Rico) territories. Most studies originated in Cuba (*n* = 7) and Jamaica (*n* = 7). Across the 8 categories of social determinants, there were a total of 15 different social determinants and 14 review endpoints, leaving 189 possible inequality relationship groups that could have been reported (Fig. 2). Only 30 (16%) of these relationship groups were reported by the 34 articles, leaving 159 relationship groups (84%) without an evidence base. There were 75 inequality relationships reported: 59 on breast cancer risk factors, 13 on breast cancer frequency, and 3 on breast cancer outcomes.

### Risk of bias of included studies

**Fig. 3:**
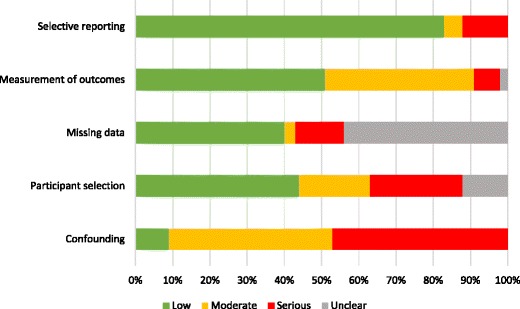
The proportion of risk of bias classifications of the 75 relationships among each of the 5 risk of bias domains [40–70, 72, 73]

Of the 34 articles, 16 were classified as moderate-risk, 14 were classified as serious-risk, 1 was classified as unclear-risk, 2 were classified as moderate/serious-risk, and 1 was classified as serious/unclear-risk (Table 2). At the relationship-level, of the 75 relationships, 35 were classified as moderate-risk, 34 were classified as serious-risk, and 6 were classified as unclear-risk. Figure 3 details the proportion of relationship classifications within each of the 5 risk of bias domains. Overall, lack of adjustment for confounding was the main contributor to an increased risk of bias, followed by non-disclosure or inadequate handling of missing data.

**Table 2:** Risk of bias among 75 relationships from 34 included articles [40–70, 72, 73]

Article (*n* = 34)	Relationship (*n* = 75)		Bias domain					
	Endpoint	Social determinant	Confounding	Participant selection	Missing data	Measurement of outcomes	Selective reporting	OVERALL
Agyemang, 2009 [46]	Overweight/obesity	Ethnicity	Serious	Low	Low	Moderate	Low	Serious
	Physical inactivity	Ethnicity	Serious	Low	Low	Low	Low	Moderate
Alvarez, 2009 [63]	Incidence	Residence	Serious	Low	Unclear	Low	Low	Moderate
Block, 2012 [40]	Alcohol	Age	Serious	Moderate	Serious	Moderate	Serious	Serious
	Physical inactivity	Age	Serious	Moderate	Serious	Moderate	Serious	Serious
Blum, 2004 [41]	Alcohol	Religion	Low	Unclear	Unclear	Moderate	Low	Unclear
Brathwaite, 2011 [47]	Overweight/obesity	Education^ind^	Serious	Moderate	Low	Low	Low	Moderate
	Overweight/obesity	Education^mat^	Serious	Moderate	Low	Low	Low	Moderate
	Overweight/obesity	Education^pat^	Serious	Moderate	Low	Low	Low	Moderate
	Overweight/obesity	Income	Serious	Moderate	Low	Low	Low	Moderate
	Overweight/obesity	Residence	Serious	Moderate	Low	Low	Low	Moderate
	Overweight/obesity	Social household structure	Serious	Moderate	Low	Low	Low	Moderate
Bryan, 2012 [48]	Overweight/obesity	Income^ins^	Serious	Low	Unclear	Serious	Low	Serious
Chatman, 2004 [60]	Breastfeeding	Age	Low	Serious	Serious	Moderate	Low	Serious
	Breastfeeding	Education	Low	Serious	Serious	Moderate	Low	Serious
	Breastfeeding	Income	Low	Serious	Serious	Moderate	Low	Serious
	Breastfeeding	Marital status	Low	Serious	Serious	Moderate	Low	Serious
	Breastfeeding	Occupation	Low	Serious	Serious	Moderate	Low	Serious
Dubois, 2011 [49]	Overweight/obesity	SEP	Low	Unclear	Low	Serious	Low	Serious
	Overweight/obesity	Social household structure	Moderate	Unclear	Low	Low	Low	Moderate
Ferguson, 2010 [50]	Overweight/obesity	Education	Moderate	Moderate	Low	Low	Low	Moderate
	Overweight/obesity	Occupation	Moderate	Moderate	Low	Low	Low	Moderate
Grievink, 2004 [51]	Overweight/obesity	Education	Moderate	Low	Low	Low	Moderate	Moderate
	Overweight/obesity	Income	Moderate	Low	Low	Low	Moderate	Moderate
	Overweight/obesity	Occupation	Moderate	Low	Low	Low	Moderate	Moderate
Hernández, 2013 [64]	Incidence	Residence	Serious	Low	Unclear	Low	Low	Moderate
Ichinohe, 2005 [52]	Overweight/obesity	Education	Moderate	Serious	Low	Low	Low	Serious
	Overweight/obesity	Marital status	Moderate	Serious	Low	Low	Low	Serious
Joseph, 2014 [65]	Incident cases	Ethnicity	Moderate	Serious	Unclear	Low	Low	Serious
	Incident cases	Marital status	Serious	Serious	Unclear	Low	Low	Serious
Kim, 2007 [42]	Alcohol	Age	Moderate	Low	Unclear	Moderate	Low	Moderate
	Alcohol	Education	Moderate	Low	Unclear	Moderate	Low	Moderate
	Alcohol	Marital status	Moderate	Low	Unclear	Moderate	Low	Moderate
	Alcohol	Residence	Moderate	Low	Unclear	Low	Low	Moderate
Laborde, 2013 [53]	Overweight/obesity	Education	Serious	Moderate	Unclear	Serious	Low	Serious
	Overweight/obesity	Income	Serious	Moderate	Unclear	Serious	Low	Serious
	Overweight/obesity	Marital status	Serious	Moderate	Unclear	Serious	Low	Serious
Latimer, 2004 [43]	Alcohol	Age	Moderate	Serious	Low	Moderate	Serious	Serious
Mendez, 2004 [54]	Overweight/obesity	Income	Moderate	Moderate	Unclear	Low	Low	Moderate
Morales, 2013 [66]	Incident cases	Education	Moderate	Low	Low	Low	Low	Moderate
	Incident cases	Marital status	Moderate	Low	Low	Low	Low	Moderate
Nam, 2012 [55]	Overweight/obesity	Education	Serious	Low	Serious	Low	Serious	Serious
	Overweight/obesity	Marital status	Serious	Low	Serious	Low	Serious	Serious
	Overweight/obesity	Residence	Serious	Low	Serious	Low	Serious	Serious
Nemesure, 2009 [67]	Incident cases	Education	Moderate	Serious	Low	Low	Low	Serious
	Incident cases	Marital status	Moderate	Serious	Low	Low	Low	Serious
	Incident cases	Occupation	Moderate	Serious	Low	Low	Low	Serious
Ohene, 2005 [44]	Alcohol	Age	Serious	Unclear	Unclear	Moderate	Low	Serious
Pérez-Ríos, 2008 [61]	Breastfeeding	Age	Moderate	Unclear	Low	Moderate	Low	Moderate
	Breastfeeding	Education	Moderate	Unclear	Low	Moderate	Low	Moderate
	Breastfeeding	Marital status	Moderate	Unclear	Low	Moderate	Low	Moderate
	Breastfeeding	Occupation	Moderate	Unclear	Low	Moderate	Low	Moderate
Rivera-Lugo, 2007 [62]	Breastfeeding	Age	Moderate	Serious	Unclear	Moderate	Low	Serious
	Breastfeeding	Education	Moderate	Serious	Unclear	Moderate	Low	Serious
	Breastfeeding	Income	Moderate	Serious	Unclear	Moderate	Low	Serious
	Breastfeeding	Marital status	Moderate	Serious	Unclear	Moderate	Low	Serious
Santana, 2011 [72]	Mortality	Residence	Serious	Low	Unclear	Low	Low	Moderate
Shirley, 2010 [68]	Incident cases	Residence	Serious	Low	Unclear	Low	Low	Moderate
Sinnapah, 2009 [56]	Overweight/obesity	Ethnicity	Serious	Low	Moderate	Unclear	Serious	Serious
	Physical inactivity	Ethnicity	Serious	Low	Moderate	Unclear	Serious	Serious
Sinnapah, 2009 [57]	Overweight/obesity	Ethnicity	Serious	Serious	Low	Moderate	Low	Serious
	Physical inactivity	Ethnicity	Serious	Serious	Low	Moderate	Serious	Serious
Sinnapah, 2009 [58]	Overweight/obesity	Ethnicity	Moderate	Low	Low	Low	Moderate	Moderate
Taioli, 2012 [73]	Mortality	Ethnicity	Moderate	Low	Unclear	Low	Low	Moderate
Torres, 2007 [69]	Incidence	Residence	Serious	Low	Unclear	Low	Low	Moderate
Torres-Cintrón, 2010	Incidence	Residence	Moderate	Low	Unclear	Low	Low	Moderate
	Mortality	Residence	Moderate	Low	Unclear	Low	Low	Moderate
Tull, 2005 [59]	Overweight/obesity	Ethnicity	Serious	Unclear	Unclear	Low	Low	Serious
van Leeuwaarde, 2011 [72]	Incidence	Ethnicity	Serious	Low	Unclear	Low	Low	Moderate
Varona, 2011 [45]	Alcohol	Age	Moderate	Low	Unclear	Moderate	Low	Moderate
	Alcohol	Education	Serious	Low	Unclear	Moderate	Low	Unclear
	Alcohol	Ethnicity	Serious	Low	Unclear	Moderate	Low	Unclear
	Alcohol	Income	Serious	Low	Unclear	Moderate	Low	Unclear
	Alcohol	Marital status	Serious	Low	Unclear	Moderate	Low	Unclear
	Alcohol	Occupation	Serious	Low	Unclear	Moderate	Low	Unclear

^ind^ – individual; ^ins^ – type of health insurance; ^mat^ – maternal; ^pat^ – paternal

## Results of inequality relationships

### Risk factors

#### Alcohol

There were 14 inequality relationships for alcohol, reported across 8 social determinants in 6 articles: age (*n* = 5), education (*n* = 2), ethnicity (*n* = 1), income (*n* = 1), marital status (*n* = 2), occupation (*n* = 1), religion (*n* = 1), residence (*n* = 1) [40–45].

All adolescent studies found that older adolescents consumed more alcohol than younger adolescents [43, 44], with less conclusive findings among adults [40, 42, 45]. Persons with higher education tended to drink more than those with less education in Barbados and Cuba [42, 45]. For example, 1.1% of elderly in Barbados with 1–6 years education versus 11.8% of persons with >12 years education consumed alcohol ≥4 days/week [42]; likewise, 4.8% (95% CI 3.8–5.7) of Cuban adults with primary level education versus 13.2% (95% CI 10.8–15.7) with university level education consumed alcohol in the past 30 days [45]. However, Cuban elderly report low frequency of consumption across all education levels (0%–1.5% consume alcohol ≥4 days/week) [42, 45]. This is in line with the one article examining residence, which reported higher frequency in overall consumption in Barbados (2.7%) as compared to Cuba (1.1%) [42]. With respect to ethnicity, more black and mestizo Cubans reported alcohol consumption (14.9%, 95% CI 12.3–17.6 and 14.7%, 95% CI 12.9, 16.5 respectively) within the past 30 days than white Cubans (8.2%, 95% CI 7.3–9.0) [45]. A large regional study found that adolescents with increased religious service attendance consumed alcohol less frequently than those who had less attendance (OR 0.50, *p* < 0.001) [41]. Studies examining marital status showed mixed findings; those examining income and occupation showed no association [42, 45].

#### Overweight/Obesity

There were 28 inequality relationships for overweight/obesity, reported across 8 social determinants in 14 articles: education (*n* = 8), ethnicity (*n* = 5), income (*n* = 5), marital status (*n* = 3), occupation (*n* = 2), residence (*n* = 2), social household structure (*n* = 2), and SEP (*n* = 1) [46–59].

Studies examining education and occupation tended towards a negative relationship [47, 50–53, 55]. All but one study (examining elderly) reported overweight/obesity to be associated with lower levels of individual education [47, 50–53], as well as maternal and paternal education [50]. Adults with lower-level occupations and children with parents working in lower-level occupations tended to be more overweight/obese than those with higher-level occupations [50, 51]. Yet reports on income showed mixed results [47, 48, 51, 53, 54], and the single study examining SEP showed higher levels of overweight/obesity among Jamaican girls of a higher family SEP (OR 1.87, 95% CI 1.0-3.4) [49]. Studies reporting on ethnicity, marital status, social household structure and residence showed mixed results.

#### Limited breastfeeding

There were 13 inequality relationships for breastfeeding, reported across 5 social determinants in 3 articles: age (*n* = 3), education (*n* = 3), income (*n* = 2), marital status (*n* = 3), and occupation (*n* = 2) [60–62].

The likelihood of breastfeeding initiation was higher among older mothers in Puerto Rico (OR 1.39, 95% CI 1.00–1.95 for 35–49 year olds), with no age differences found in Jamaica [60, 61]. Also, Puerto Rican mothers who practiced breastfeeding initiation and exclusive breastfeeding tended to be more educated than those who did not [61, 62]. Mixed results were found for marital status, income, and occupation; to note is that Jamaican mothers who were employed were less likely to exclusively breastfeed (of those employed, 21.1% exclusively breastfeed versus 31.0% nonexclusively breastfeed), while Puerto Rican mothers who were employed were more likely to initiate breastfeeding (crude OR 1.63, 95% CI 1.31–2.03; adjusted OR 1.15, 95% CI 0.89–1.48) [60, 61].

#### Physical inactivity

There were 4 inequality relationships for physical inactivity, reported across 2 social determinants in 4 articles: age (*n* = 1), ethnicity (*n* = 3) [40, 46, 56, 57].

In Grenada, the amount of persons participating in physical activity through walking/biking drastically decreased by 72.5% (*p* = <0.001) after 54 years of age; at the same time, the amount of persons participating in >10 min of leisure time per day was also found to gradually increase with age (78.1% for persons <35 years old to 83.5% for persons >64 years old, *p* = 0.53) [40]. The two studies examining ethnicity found that Guadeloupian Asian-Indian adults reported lower levels physical activity than their non-Asian-Indian counterparts when considering time and level of vigour of activity (physical activity level score mean 1.62 (SD 0.22) versus mean 1.74 (SD 0.34), *p* = <0.05) [56, 57].

#### Frequency & outcomes

Fewer studies examined the social determinants of the frequency and outcomes of breast cancer, than those for risk factors. There were 13 inequality relationships for breast cancer frequency, reported across 5 social determinants in 9 articles: education (*n* = 2), ethnicity (*n* = 2), marital status (*n* = 3), occupation (*n* = 1), and residence (*n* = 5) [63–71]. Most articles reported the number of new breast cancer cases, with 4 out of the 9 articles converting these counts to a breast cancer incidence rate. Relationships examining occupation, residence, and ethnicity showed no association. A Puerto Rico study found a higher likelihood of breast cancer among women with only primary and secondary education as compared to women with higher education (OR 3.38, 95% CI 1.5–5.7 for primary; OR 1.33, 95% CI 0.9–1.9 for secondary) [66]. Lastly, unmarried women in Puerto Rico tended to have a higher likelihood of being diagnosed with breast cancer as compared to married women (divorced OR 2.57, 95% CI 1.4–4.4; single OR 1.36, 95% CI 0.7–2.6; widow OR 2.08, 95% CI 1.1–4.0) [66], but no differences were seen in Trinidad or Barbados.

There were 3 inequality relationships for breast cancer mortality, reported across 2 social determinants in 3 articles: ethnicity (*n* = 1) and residence (*n* = 2) [72, 73]. No evidence found reporting on the other 4 breast cancer outcomes. While no associations were observed between breast cancer frequency and ethnicity, mortality from breast cancer was shown to be higher among Indian-decent compared to African-descent populations in Trinidad (OR 1.2, 95% CI 1.1–1.4) and Guyana (OR 1.3, 95% CI 1.0–1.6) [73].

## Discussion

### Summary of evidence

This systematic review examined the extent of evidence on the influence of social determinants of health on breast cancer risk factors, frequency, and adverse outcomes in the Caribbean. Thirty-four articles from 32 separate studies were included. With 189 possible ways of exploring the role of social determinants on breast cancer, 75 inequality relationships were reported within 30 distinct relationship groups, leaving 159 (84%) relationship groups without an evidence base. The results of this review highlight a critical evidence gap on the effects of social determinants on breast cancer among Caribbean women, with limitations in the quantity and quality of published evidence. Nearly half of the articles were classified as having serious risk of bias, mostly because of failure to adjust for important potential confounders. Furthermore, included articles reported a range of inconclusive findings for each relationship group, at least partly due to study heterogeneity and small numbers of studies available for each relationship group.

Measures of breast cancer frequency and adverse outcomes showed weak relationships with social determinants. Though, the racial disparity in breast cancer mortality between women of Indian origin and women of African origin in two different settings is worthy further investigation. The connection between breast cancer and social inequity is a not a new phenomenon. While low social status is known to place women at a higher risk of developing and dying from breast cancer [74, 75], a higher social status tends to predispose women to certain reproductive risk factors including later age at first pregnancy, lower parity and less breastfeeding [76, 77]. However, a higher SEP also affords women a higher screening rate, an earlier stage of diagnosis, and improved treatment effect and adherence, indicating a complex interchange of risk and protection [74, 75, 78]. Our depicted lack of regional evidence seems a logical result of the absence of a structured network of cancer surveillance in the Caribbean [79, 80]. Cancer registries exist in only twelve Caribbean territories, of which only four are considered high-quality [80, 81]. Challenges are wide-reaching, with limitations in resources, political will, policy and regulation, healthcare service, data quality and security, and local, regional, and international communication and collaboration [80, 81]. The PAHO Plan of Action for Cancer Prevention and Control 2008–2015 [82] has detailed areas for improvement in monitoring and surveillance and consequently, the regional Caribbean Cancer Registry Hub was conceptualized and is progressing towards implementation [81]. While this Hub is expected to greatly improve regional cancer surveillance efforts, measures of inequalities should be highlighted in its plans, with hopes to increase attention to social determinants of cancers and advance health promotion in this area.

Most results lie within the relationships between social determinants and breast cancer risk factors. Overall, Caribbean women with indicators of a lower SEP could be at a higher risk of breast cancer as they reported a higher alcohol intake (except for education), higher levels of overweight/obesity, and limited breastfeeding. The trends reported between age and education with breastfeeding is in line with evidence in other settings, with low maternal education being the strongest predictor of poor breastfeeding practices [83–86]. The inverse relationship between overweight/obesity and education and occupation is similar to what is found in other middle and higher income regions; while being overweight or obese was previously thought to be a condition of the elite, more recent transitions have occurred whereby obesity is shifting towards the persons with a lower socioeconomic standing, particularly as the country’s gross national product increases [87–89]. Typically though, alcohol consumption is found to be higher among persons of a higher SEP [90, 91]. Yet the relationship between alcohol and SEP is complex. Varying environmental factors such as alcohol availability and affordability, economic development, culture, and national alcohol policy flout the gradient typically observed whereby risk factor harm increases with decreasing SEP [90, 91]. The Caribbean is particularly vulnerable to this risk factor as its cultural norms embrace alcohol consumption as a commonplace social activity, which is further compounded by a lack of national alcohol policies [92–94]. While no relationships were reported on social capital, the inverse relationship between alcohol and religion is noteworthy. Religiosity is consistently shown to be protective from substance use by creating a positive personal identity, fostering community acceptance, and providing a coping outlet for stress [95–97]. The Caribbean touts a predominant religious identity which could confer some form of protection from alcohol’s influence on breast cancer and the wider range of NCDs afflicting the region.

Continued and standardized approaches to understanding risk factor profiles is a key element in efforts to reduce cancer risk factors, as evidenced in the WHO’s recommended STEPwise approach to Surveillance (STEPS) [98]. With relevant information on social determinants included in this instrument, it is up to Caribbean territories to fulfil their commitment to the Port-of-Spain Declaration in continuing to implement this in their ongoing efforts to reduce NCDs such as breast cancer [99].

### Limitations

The review was limited by a small number of articles within each relationship group, the validity of which was further limited by their significant risk of bias. Further, few studies investigating the effects of social determinants on health have also explored the interrelationships among the social determinants themselves. The Caribbean has been considered as one region in this review, masking the possible and important country-level variations in the relative importance of social determinants. Country-level information on screening and access to treatment such as mammogram screening rates and wait times for diagnosis or treatment are important potential confounders that were not assessed. Publication bias is an important concern as no explicit searching was conducted for grey literature due to limited resources.

## Conclusions

This review highlights a crucial gap in the quantity and quality of the evidence examining the social determinants of breast cancer risk factors, frequency, and outcomes. Risk factors were the main endpoints for which relationships with social determinants were reported, with implications for age, ethnicity, education, SEP, and religion. Information on frequency and outcomes were limited, but held implications on marital status and ethnicity respectively. Although the need for more research in this area is acknowledged, this effort should also include an attempt at standardizing reporting guidelines for observational studies of health inequality. Finally, the development of a validated risk of bias assessment tool is imperative for systematic reviewing of observational studies.

## Additional files

Additional file 1: “Study Protocol”, which details the study protocol for the systematic review. (PDF 1800 kb)

Additional file 2: “Search Strategy”, which details the search strategies of the database. (PDF 469 kb)

## Abbreviations

CINAHL: Cumulative Index of Nursing and Allied Health Literature
CSDH: Commission on the Social Determinants of Health
CUMED: Cuba Medicina
EMBASE: Excerpta Medica Database
IBECS: Índice Bibliográfico Español en Ciencias de la Salud
LILACS: Latin American and Caribbean Health Sciences
MEDLINE: Medical Literature Analysis and Retrieval System Online, or MEDLARS Online
NCD: Non-communicable disease
SciELO: Scientific Electronic Library Online
SEP: Socioeconomic position
STROBE: Strengthening the reporting of observational studies in epidemiology
USCAHDR: United States Caribbean Alliance for Health Disparities Research Group
WHO: World Health Organization
