# Supplementary material for: Social determinants of breast cancer in the Caribbean: a systematic review
Source: Int J Equity Health. 2017 Apr 5;16:60. doi: 10.1186/s12939-017-0540-z (PMC5382386; doi:10.1186/s12939-017-0540-z)
Supplement: Supplementary file 2 — “Search Strategy”, which details the search strategies of the database. (PDF 469 kb) [file 12939_2017_540_MOESM2_ESM.pdf]

Search Strategy

Database: Medline
Search Engine: Pubmed
Date Searched: 31/12/2014
Filters Used: human only; dates 01/01/2004 – 31/12/2014 only
Number of Results: 3880
Notes: We restricted words that could be author names to non-author fields. We don't use (*) to truncate words in cases where the non-truncated word is actually a broader search because it triggers a MeSH term and automatically includes the pluralized form, or we include both the truncated and non-truncated (MeSH) terms.

Search Strategy: (Caribbean OR West Indies OR Leeward OR Windward OR Antilles OR Anguilla OR Antigua OR Aruba OR Barbuda OR Bahamas OR Barbados OR Barthelemy OR "St. Bartholomew" OR "Saint Bartholomew" OR Barts OR Belize OR Bermuda OR Bonaire OR Cayman OR Croix OR Cuba OR Curacao OR Dominica OR "Dominican Republic" OR Eustatius OR "Santo Domingo" OR "Saint Domingue" OR "St. Domingue" OR Grenada OR Guadeloupe OR Guyana OR Haiti OR Hispaniola OR Jamaica OR "St. John" OR "Saint John" OR "St. Thomas" OR "Saint Thomas" OR "St. Vincent" OR "Saint Vincent" OR "St. Martin" OR "Saint Martin" OR "St. Maarten" OR "Saint Maarten" OR Martinique[tw] OR Martinique[AD] OR Martinique [TA] OR Martinique [LID] OR Martinique [PL] OR Martinique [PUBN] OR "St. Nevis" OR "Saint Nevis" OR "St. Christopher and Nevis" OR "Saint Christopher and Nevis" OR "St. Lucia" OR "Saint Lucia" OR Kitts OR Montserrat OR "Puerto Rico" OR Grenadines OR "Virgin Islands" OR Saba OR Suriname OR Trinidad OR Tobago OR Tortola) AND (age OR gender OR education OR educat*OR income OR wealth OR ethnic OR ethnic* OR race OR culture OR language OR occupation OR religion OR social class OR socioeconomic OR health social determinants OR social determinant* OR social capital OR residence OR medical geography OR health service OR health service* OR health equity OR disparit* OR medical sociology OR prejudice OR health insurance OR health gradient OR health gap OR vulnerable populations OR continental population groups OR Arawak* OR Amerindian* OR carib OR caribs OR taino* OR ethnic groups OR social conditions OR urban OR rural OR urban health OR urban population OR rural health OR rural population OR social position OR poverty OR wealth OR rich[tw] OR poor OR social support OR discriminat* OR differenti* OR globaliz* OR globalis* OR urbanization OR urbaniz* OR urbanis* OR westerniz* OR westernis*) AND ("Breast Neoplasms" [MeSH] OR ((breast OR ductal OR lobular OR (mammary AND gland)) AND (cancer OR sarcoma OR carcinoma OR tumor OR neoplasm OR mucosa associated lymphoid tissue OR MALT OR stage OR stages OR staging OR grade OR grades OR grading OR recurren* OR remission OR (year AND survival))) OR (Alcohol OR drinking or Bmi OR body mass index OR overweight OR obesity OR obes* OR sugar OR sweet* OR (Physical* AND (active* OR activi* OR inactiv*)) OR motor activity OR exercise OR radiation OR x-ray OR (age AND first AND (pregnancy OR pregnant OR birth)) OR maternal age OR Parity OR parous OR (Number AND (children OR live births OR babies OR delivered OR deliveries)) OR breastfeed OR breastfeeding OR breastfed))

Database: SciELO
Search Engine: SciELO
Date Searched: 31/12/2014
Filters Used: none (option not available)
Number of Results: 745
Notes: We eliminated social determinants from the search strategy for SciELO because this search engine doesn't seem very sensitive and didn't pick up on social determinants that were in the abstract of papers we would like to include. Note also that we use (*) here since SciELO doesn't automatically map to pluralized forms of search terms. Also we added words with multiple spellings, ie tumor OR tumuor since SciELO doesn't automatically do this.

Search Strategy: (Caribbean OR "West Indies"OR Leeward OR Windward OR Antilles OR Anguilla OR Antigua OR Aruba OR Barbuda OR Bahamas OR Barbados OR Barthelemy OR "St. Bartholomew" OR "Saint Bartholomew" OR Barts OR Belize OR Bermuda OR Bonaire OR Cayman OR Croix OR Cuba OR Curacao OR Dominica OR "Dominican Republic" OR Eustatius OR "Santo Domingo" OR "Saint Domingue" OR "St. Domingue" OR Grenada OR Guadeloupe OR Guyana OR Haiti OR Hispaniola OR Jamaica OR "St. John" OR "Saint John" OR "St. Thomas" OR "Saint Thomas" OR "St. Vincent" OR "Saint Vincent" OR "St. Martin" OR "Saint Martin" OR "St. Maarten" OR "Saint Maarten" OR Martinique OR "St. Nevis" OR "Saint Nevis" OR "St. Christopher and Nevis" OR "Saint Christopher and Nevis" OR "St. Lucia" OR "Saint Lucia" OR Kitts OR Montserrat OR "Puerto Rico" OR Grenadines OR "Virgin Islands" OR Saba OR Suriname OR Trinidad OR Tobago OR Tortola) AND (((breast OR ductal OR lobular OR (mammary AND gland)) AND (cancer* OR sarcoma* OR carcinoma* OR tumor* OR tumour*OR neoplasm* OR "mucosa associated lymphoid tissue" OR MALT OR stage OR stages OR staging OR grade OR grades OR grading OR recurren* OR remission OR (year AND survival))) OR (Alcohol OR drinking or Bmi OR "body mass index" OR overweight OR obes* OR sugar* OR sweet* OR (Physical* AND (active OR activi* OR inactiv*)) OR "motor activity" OR exercise OR radiation OR x-ray OR (age AND first AND (pregnancy OR pregnant OR birth)) OR "maternal age" OR Parity OR parous OR (Number AND (children OR "live births" OR babies OR delivered OR deliveries)) OR breastfeed OR breastfeeding OR breastfed))

Database: CINHAL
Search Engine: EBSCO
Date Searched: 31/12/2014
Filters Used: human only; dates 01/01/2004 – 31/12/2014 only
Number of Results: 635
Notes: We use (*) here since CINAHL doesn't automatically map to pluralized forms of search terms. Also we added tumo#r to capture both tumor and tumour. To mirror the restrictions we put on words that could be author names for our PubMed search, we used the following search field abbreviations to limit search results to non-author fields – note that we do this for island names, and for the social determinant "rich"

Search Strategy: (Caribbean OR "West Indies" OR Leeward OR Windward OR Antilles OR Anguilla OR Antigua OR Aruba OR Barbuda OR Bahamas OR Barbados OR Barthelemy OR "St. Bartholomew" OR "Saint Bartholomew" OR Barts OR Belize OR Bermuda OR Bonaire OR Cayman OR Croix OR Cuba OR Curacao OR Dominica OR "Dominican Republic" OR Eustatius OR "Santo Domingo" OR "Saint Domingue" OR "St. Domingue" OR Grenada OR Guadeloupe OR Guyana OR Haiti OR Hispaniola OR Jamaica OR "St. John" OR "Saint John" OR "St. Thomas" OR "Saint Thomas" OR "St. Vincent" OR "Saint Vincent" OR "St. Martin" OR "Saint Martin" OR "St. Maarten" OR "Saint Maarten" OR Martinique OR "St. Nevis" OR "Saint Nevis" OR "St. Christopher and Nevis" OR "Saint Christopher and Nevis" OR "St. Lucia" OR "Saint Lucia" OR Kitts OR Montserrat OR "Puerto Rico" OR Grenadines OR "Virgin Islands" OR Saba OR Suriname OR Trinidad OR Tobago OR Tortola) AND (age OR gender OR educat* OR income OR wealth OR ethnic* OR race OR culture OR language OR occupation OR religion OR "social class" OR socioeconomic OR "social determinant" OR "social determinants" OR "social capital" OR residence OR "health service" OR "health services" OR "health equity" OR disparit* OR prejudice OR "health insurance" OR "health gradient" OR "health gap" OR "vulnerable population" OR "vulnerable populations" OR Arawak* OR Amerindian* OR carib OR caribs OR taino* OR urban OR rural OR poverty OR wealth OR rich OR poor OR "social support" OR discriminat* OR differenti* OR globaliz* OR globalis* OR urbanization OR urbaniz* OR urbanis* OR westerniz* OR westernis*) AND (((breast OR ductal OR lobular OR (mammary AND gland)) AND (cancer* OR sarcoma* OR carcinoma* OR tumor* OR tumour* OR neoplasm* OR mucosa associated lymphoid tissue OR MALT OR stage OR stages OR staging OR grade OR grades OR grading OR recurren* OR remission OR (year AND survival))) OR (Alcohol OR drinking or Bmi OR body mass index OR overweight OR obesity OR obes* OR sugar* OR sweet* OR (Physical* AND (active* OR activi* OR inactiv*)) OR motor activity OR exercise OR radiation OR x-ray OR (age AND first AND (pregnancy OR pregnant OR birth)) OR maternal age OR Parity OR parous OR (Number AND (children OR live births OR babies OR delivered OR deliveries)) OR breastfeed OR breastfeeding OR breastfed))

Database: LILACS, CUMED, IBEDS
Search Engine: WHO Virtual Health Library
Date Searched: 31/12/2014
Filters Used: human only; dates 01/01/2004 – 31/12/2014 only; Medline excluded; LILACS, CUMED, IBEDS databases only
Number of Results: 600
Notes: We use (*) here since VHL doesn't automatically map to pluralized forms of search terms. Also we added tumor and tumour. It wasn't obvious how to use search field abbreviations, so we used the drop down menu in the advanced search tool to manually broaden our search for geography. Note also that we can use "$" or "*" for truncation symbols in VHL. Note that we have to add parantheses around each phrase, and also replace the quotation marks with (. Note, exclude Medline in database filters, and restrict to human and year. We need to save the database that this draws upon and what number of hits are attributed to each database.

Search Strategy: (Caribbean OR (West Indies) OR Leeward OR Windward OR Antilles OR Anguilla OR Antigua OR Aruba OR Barbuda OR Bahamas OR Barbados OR Barthelemy OR (St. Bartholomew) OR (Saint Bartholomew) OR Barts OR Belize OR Bermuda OR Bonaire OR Cayman OR Croix OR Cuba OR Curacao OR Dominica OR (Dominican Republic) OR Eustatius OR (Santo Domingo) OR (Saint Domingue) OR (St. Domingue) OR Grenada OR Guadeloupe OR Guyana OR Haiti OR Hispaniola OR Jamaica OR (St. John) OR (Saint John) OR (St. Thomas) OR (Saint Thomas) OR (St. Vincent) OR (Saint Vincent) OR (St. Martin) OR (Saint Martin) OR (St. Maarten) OR (Saint Maarten) OR Martinique OR (St. Nevis) OR (Saint Nevis) OR (St. Christopher and Nevis) OR (Saint Christopher and Nevis) OR (St. Lucia) OR (Saint Lucia) OR Kitts OR Montserrat OR (Puerto Rico) OR Grenadines OR (Virgin Islands) OR Saba OR Suriname OR Trinidad OR Tobago OR Tortola) AND (age OR gender OR educat* OR income OR wealth OR ethnic* OR race OR culture OR language OR occupation OR religion OR (social class) OR socioeconomic OR (social determinant) OR (social determinants) OR (social capital) OR residence OR (health service) OR (health services) OR (health equity) OR disparit* OR prejudice OR (health insurance) OR (health gradient) OR (health gap) OR (vulnerable population) OR (vulnerable populations) OR Arawak* OR Amerindian* OR carib OR caribs OR taino* OR urban OR rural OR poverty OR wealth OR rich OR poor OR (social support) OR discriminat* OR differenti* OR globaliz* OR globalis* OR urbanization OR urbaniz* OR urbanis* OR westerniz* OR westernis*) AND (((breast OR ductal OR lobular OR (mammary AND gland)) AND (cancer* OR sarcoma* OR carcinoma* OR tumor* OR tumour*OR neoplasm* OR (mucosa associated lymphoid tissue) OR MALT OR stage OR stages OR staging OR grade OR grades OR grading OR recurren* OR remission OR (year AND survival))) OR (Alcohol OR drinking or Bmi OR (body mass index) OR overweight OR obesity OR obes* OR sugar* OR sweet* OR (Physical* AND (active* OR activi* OR inactiv*)) OR (motor activity) OR exercise OR radiation OR x-ray OR (age AND first AND (pregnancy OR pregnant OR birth)) OR (maternal age) OR Parity OR parous OR (Number AND (children OR (live births) OR babies OR delivered OR deliveries)) OR breastfeed OR breastfeeding OR breastfed))

Database: EMBASE
Search Engine: Ovid
Date Searched: 25/02/2015
Filters Used: human only; dates 01/01/2004 – 31/12/2014 only; Medline excluded
Number of Results: 574
Notes: This is the same as VHL, but using ' ' instead of ( ); we also had to take out (St. Christopher and Nevis) because it was interfering with the search results.

Search Strategy: (Caribbean OR 'West Indies' OR Leeward OR Windward OR Antilles OR Anguilla OR Antigua OR Aruba OR Barbuda OR Bahamas OR Barbados OR Barthelemy OR 'St. Bartholomew' OR 'Saint Bartholomew' OR Barts OR Belize OR Bermuda OR Bonaire OR Cayman OR Croix OR Cuba OR Curacao OR Dominica OR 'Dominican Republic' OR Eustatius OR 'Santo Domingo' OR 'Saint Domingue' OR 'St. Domingue' OR Grenada OR Guadeloupe OR Guyana OR Haiti OR Hispaniola OR Jamaica OR 'St. John' OR 'Saint John' OR 'St. Thomas' OR 'Saint Thomas' OR 'St. Vincent' OR 'Saint Vincent' OR 'St. Martin' OR 'Saint Martin' OR 'St. Maarten' OR 'Saint Maarten' OR Martinique OR 'St. Nevis' OR 'Saint Nevis' OR 'St. Lucia' OR 'Saint Lucia' OR Kitts OR Montserrat OR 'Puerto Rico' OR Grenadines OR 'Virgin Islands' OR Saba OR Suriname OR Trinidad OR Tobago OR Tortola) AND (age OR gender OR educat* OR income OR wealth OR ethnic* OR race OR culture OR language OR occupation OR religion OR (social class) OR socioeconomic OR 'social determinant' OR 'social determinants' OR 'social capital' OR residence OR 'health service' OR 'health services' OR 'health equity' OR disparit* OR prejudice OR 'health insurance' OR 'health gradient' OR 'health gap' OR 'vulnerable population' OR 'vulnerable populations' OR Arawak* OR Amerindian* OR carib OR caribs OR taino* OR urban OR rural OR poverty OR wealth OR rich OR poor OR 'social support' OR discriminat* OR differenti* OR globaliz* OR globalis* OR urbanization OR urbaniz* OR urbanis* OR westerniz* OR westernis*) AND (((breast OR ductal OR lobular OR (mammary AND gland)) AND (cancer* OR sarcoma* OR carcinoma* OR tumor* OR tumour*OR neoplasm* OR 'mucosa associated lymphoid tissue' OR MALT OR stage OR stages OR staging OR grade OR grades OR grading OR recurren* OR remission OR (year AND survival))) OR (Alcohol OR drinking or Bmi OR 'body mass index' OR overweight OR obesity OR obes* OR sugar* OR sweet* OR (Physical* AND (active* OR activi* OR inactiv*)) OR 'motor activity' OR exercise OR radiation OR x-ray OR (age AND first AND (pregnancy OR pregnant OR birth)) OR 'maternal age' OR Parity OR parous OR (Number AND (children OR (live births) OR babies OR delivered OR deliveries)) OR breastfeed OR breastfeeding OR breastfed))
